# Two concurrent outbreaks of hepatitis A highlight the risk of infection for non-immune travellers to Morocco, January to June 2018

**DOI:** 10.2807/1560-7917.ES.2018.23.27.1800329

**Published:** 2018-07-05

**Authors:** Martyna Gassowski, Kai Michaelis, Jürgen J. Wenzel, Mirko Faber, Julie Figoni, Lina Mouna, Ingrid HM Friesema, Harry Vennema, Ana Avellon, Carmen Varela, Lena Sundqvist, Josefine Lundberg Ederth, James Plunkett, Koye Balogun, Siew Lin Ngui, Sofie Elisabeth Midgley, Sofie Gillesberg Lassen, Luise Müller

**Affiliations:** 1 These authors contributed equally to this work; 2 Department for Infectious Disease Epidemiology, Robert Koch Institute (RKI), Berlin, Germany; 3 Postgraduate Training for Applied Epidemiology (PAE, German Field Epidemiology Training Programme), Robert Koch Institute, Berlin, Germany; 4 European Programme for Intervention Epidemiology Training (EPIET), European Centre for Disease Prevention and Control (ECDC), Stockholm, Sweden; 5 Department for Infectious Disease Epidemiology, Unit of Gastrointestinal Infections, Zoonoses, and Tropical Infections, Robert Koch Institute (RKI), Berlin, Germany; 6 National Consultant Laboratory for HAV and HEV, Institute of Clinical Microbiology and Hygiene, University Medical Centre Regensburg, Regensburg, Germany; 7 Santé Publique France, French National Public Health Agency, Saint-Maurice, France; 8 AP-HP, National Reference Centre for Enterically Transmitted Hepatitis Viruses, Paul Brousse hospital, Villejuif, France.; 9 Centre for Infectious Diseases, Epidemiology and Surveillance, Centre for Infectious Disease Control, National Institute for Public Health and the Environment (RIVM), Bilthoven, the Netherlands; 10 Centre for Infectious Diseases Research, Diagnostics and Screening, Centre for Infectious Disease Control, National Institute for Public Health and the Environment (RIVM), Bilthoven, the Netherlands; 11 Hepatitis Unit, National Center of Microbiology, Instituto de Salud Carlos III, Madrid, Spain; 12 National Centre of Epidemiology, Instituto de Salud Carlos III, CIBER Epidemiología y Salud Pública, Madrid, Spain; 13 Department of Communicable Disease Control and Health Protection, the Public Health Agency of Sweden, Stockholm, Sweden; 14 Department of Microbiology, the Public Health Agency of Sweden, Stockholm, Sweden; 15 National Infection Service, Public Health England, London, United Kingdom; 16 Department of Virus & Microbiological Special Diagnostics, Statens Serum Institut, Copenhagen, Denmark; 17 Department of Infectious Disease Epidemiology and Prevention, Statens Serum Institut, Copenhagen, Denmark

**Keywords:** hepatitis A, hepatitis A virus, food-borne infections, outbreaks, travel, imported viral diseases

## Abstract

From January to June 2018, two ongoing hepatitis A outbreaks affected travellers returning from Morocco and cases in Europe without travel history, resulting in 163 patients in eight European countries. Most interviewed travel-related cases were unaware of the hepatitis A risk in Morocco. Molecular analysis revealed two distinct hepatitis A virus (HAV) strains (subgenotype IA DK2018_231; subgenotype IB V18–16428). Vaccination recommendations should be emphasised to increase awareness among non-immune travellers to Morocco and HAV-endemic countries.

We report on two distinct hepatitis A virus strains that are causing cases in travellers returning from Morocco and autochthonous cases in several European countries between 1 January and 18 June 2018.

## The alert

On 2 May 2018, Denmark reported a cluster of hepatitis A virus (HAV) infections with the subgenotype IA strain DK2018_231, through the European Centre for Disease Prevention and Control (ECDC)’s Epidemic Intelligence Information System (EPIS) for food- and waterborne diseases and zoonoses (FWD). One of the three confirmed cases had travelled to Morocco. In response, five additional European Union (EU) countries (France, Germany, the Netherlands, Spain and the United Kingdom (UK)) reported cases (n = 20) infected with the same strain between 21 January and 10 April 2018. Concurrently, Germany reported to EPIS that it observed more cases of hepatitis A with travel history to Morocco than expected, compared with the same period in the previous 5 years. Molecular analysis of the HAV VP1/P2A region revealed an unrelated cluster of the HAV subgenotype IB strain V18–16428. Cases infected with this unrelated strain were also reported from France, the Netherlands, Sweden and UK.

The appearance of clusters with a link to Morocco triggered further epidemiological investigations.

## Case definitions and outbreak curve

For the investigation of the two clusters, the case definitions in the Box were used. The Figure shows the epidemiological curve depicting both clusters.

Box **Case definitions for confirmed travel-related or autochthonous hepatitis A cases infected with the subgenotype IA virus strain DK2018_231 or the subgenotype IB virus strain V18–16428**

**Table:** 

**Travel-related confirmed cases**	
• An EU/EEA resident with laboratory-confirmed hepatitis A and date of symptoms onset (or date of sampling if onset date not available or if the case is asymptomatic) on or after 1 January 2018 and • with a travel history to Morocco in the 50 days before symptoms onset And	
*Cluster with subgenotype IA*	*Cluster with subgenotype IB*
• a sequence with ≥ 99.4% identity to the HAV subgenotype IA outbreak strain DK2018_231, based on an overlapping fragment at the VP1/P2A region.	• a sequence with ≥ 99.4% identity to the HAV subgenotype IB outbreak strain V18–16428, based on an overlapping fragment at the VP1/P2A region.
**Autochthonous confirmed cases**	
• An EU/EEA resident with laboratory-confirmed hepatitis A and date of symptoms onset (or date of sampling if onset date not available or if the case is asymptomatic) on or after 1 January 2018 and • without a travel history out of the EU/EEA in the 50 days before symptoms onset And	
*Cluster with subgenotype IA*	*Cluster with subgenotype IB*
• a sequence with ≥ 99.4% identity to the HAV subgenotype IA outbreak strain DK2018_231, based on an overlapping fragment at the VP1/P2A region	• a sequence with ≥ 99.4% identity to the HAV subgenotype IB outbreak strain V18–16428, based on an overlapping fragment at the VP1/P2A region
**Travel-related possible cases**	
• An EU/EEA resident with laboratory-confirmed hepatitis A and date of symptoms onset (or date of sampling if onset date not available or if the case is asymptomatic) on or after 1 January 2018 and • with a travel history to Morocco in the 50 days before symptoms onset and • without sequencing characterisation of the viral RNA.	

EU/EEA: European Union/European Economic Area; HAV: hepatitis A virus.In order to consider possible mutations, we included closely related strains with a cut-off of 2 nucleotides (99.4% identity) in the case definition.

**Figure:**
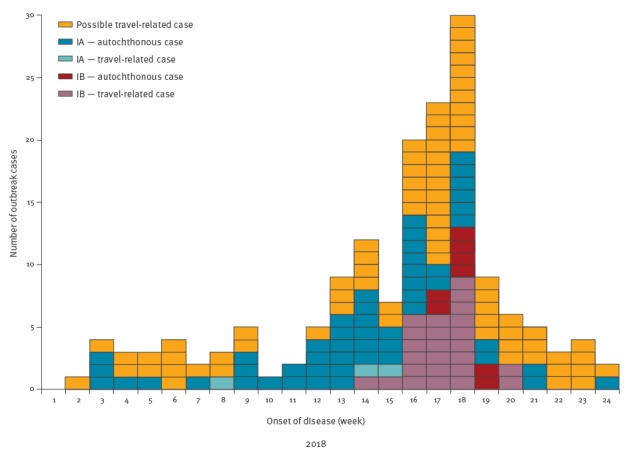
Number of hepatitis A virus cases by case definition, hepatitis A virus subgenotype cluster and week of disease onset, 1 January–18 June 2018 (n = 163)

IA: subgenotype IA, strain DK2018_231; IB: subgenotype IB strain V18–16428.

## Subgenotype IA cluster (DK2018_231)

As at 18 June 2018, 55 cases belonging to the subgenotype IA-cluster were reported from eight European countries. The UK reported the highest number of cases (n = 36). Denmark, France, Germany, Ireland, the Netherlands, Spain and Sweden each reported between one and six cases. Some few cases (n = 22/55) identified in Ireland, Spain and the UK differ from strain DK2018_231 by one or two nucleotides in the VP1/P2A junction.

Onset of symptoms among the cases ranged between mid-January and mid-June 2018. Cases were between 3 and 81 years old with a median of 31 years (interquartile range (IQR): 12.5–52 years); 47% (n = 26/55) were female. Three cases reported travel to Morocco within 50 days before disease onset; one case in their early 50s who had no known pre-existing conditions, died as a result of HAV infection.

Variants of strain DK2018_231 differing by only one or two nucleotides are considered as part of this cluster. The occurrence of closely related strains of HAV have been observed in other major food-borne outbreaks [1-3]. It is, however, still possible that cases actually belong to separate transmission chains.

## Subgenotype IB cluster (V18–16428)

As at 18 June 2018, the subgenotype IB-cluster consisted of 33 confirmed cases; 25 travel-related and eight autochthonous (Table). Most cases were reported by Germany (n = 15), France (n = 8) and the UK (n = 6), with Sweden and the Netherlands only reporting one and three cases, respectively. Sequencing results of the VP1/P2A junctions revealed a 100% identity. Onset of symptoms ranged from the beginning of April to mid-May. All travel-related cases reported travel to Morocco in March. The overall age range of cases were between 8 and 76 years, with a median of 51 years (IQR: 26–61 years); 55% (n = 18/33) were female

**Table:** Number of travel-related and autochthonous cases of hepatitis A, by country of origin and hepatitis A virus subgenotype, 1 January–18 June 2018 (n = 163)

	Number of confirmed cases, subgenotype IA, DK2018_231		Number of confirmed cases, subgenotype IB, V18–16428		Number of possible cases
Country of origin	*Travel-related*	*Autochthonous*	*Travel-related*	*Autochthonous*	*Travel-related*
Denmark	1	5	0	0	2
France	1	0	6	2	43
Germany	1	0	9	6	15
Ireland	0	1	0	0	0
The Netherlands	0	4	3	0	1
Spain	0	5	0	0	14
Sweden	0	1	1	0	0
UK	0	36	6	0	0
**Total**	**3**	**52**	**25**	**8**	**75**

UK: United Kingdom.

## Early results from case interviews in Germany and France

The travel-related cases in Germany were initially suspected to belong to the IA cluster, but the sequencing results revealed a different subgenotype. A case–control study of the German cases is underway, and 16 travel-related cases (8 confirmed, 8 possible) and four autochthonous cases have been interviewed so far for potential exposures. All autochthonous cases have reported consumption of food items brought home by travellers returning from Morocco, with dates being the only food item reported by all four cases. Among the 16 travel-associated cases, 14 reported date consumption during their stay in Morocco. In France, one patient reported bringing dates back from Morocco and eating these together with two other persons, all three of whom developed symptoms after the same incubation time.

Based on these preliminary results, dates are currently the suspected vehicle in this cluster, though investigation is ongoing.

## Vaccination status

Information regarding hepatitis A vaccination status was collected from the German travel-related cases via interview, including reasons for non-vaccination. Preliminary results show that of the 15 travel-related cases (one case refused to be re-interviewed), 13 were either not vaccinated or unsure of their vaccination status. Eleven cases stated that they had been unaware of the risk of HAV in Morocco, while six reported that they did not know there was a vaccination against hepatitis A. During the interviews, several cases stated that there was a lack of information regarding vaccination recommendations for Morocco in the travel guides and tour operator documents they had used when preparing for their trip.

Unvaccinated travel-related cases were also reported from Denmark (n = 3), France (n = 41), the Netherlands (n = 3) and Spain (n = 5); reasons for non-vaccination were not collected. Vaccination status was not available for all other travel-related cases from these countries or from Ireland, Sweden and the UK.

## Discussion and conclusion

The occurrence of the two concurrent HAV clusters in the first 6 months of 2018 serve as a reminder of the risk of contracting hepatitis A in Morocco, a country with intermediate endemicity [4,5]. HAV subgenotypes IA and IB are known to circulate in Morocco and strain DK2018_231 has been observed in sporadic cases with travel history to Morocco in previous years [6-8]. Despite the different characteristics of the two reported clusters, cases with a travel history to Morocco feature in both. In a recent study of European travellers, Turkey, Egypt and Morocco were listed as the top three destinations for acquiring travel-associated hepatitis A and accounted for one third of cases in the period 2009–15 [9]. The epidemiological link to Morocco is more apparent in cluster IB, where the majority of cases had confirmed travelling to Morocco and all interviewed autochthonous cases had reported consuming food items brought home from there.

In the IA cluster, only three cases had travelled to Morocco. However, the large proportion of autochthonous cases and their spatial distribution in this cluster suggest that an imported food item may have served as the vehicle in this outbreak. Large food-borne hepatitis A outbreaks from frozen berries and semi-dried tomatoes have previously affected European countries, further indicating that imported contaminated food products pose a risk to the increasingly susceptible general population in Europe [10-13].

The outbreaks described here illustrate the increased risk that non-immune travellers face when visiting HAV-endemic areas like Morocco. All of the eight countries where cases occurred have explicit recommendations of hepatitis A vaccination for travel to endemic countries, in accordance with World Health Organization (WHO) recommendations [14,15]. Yet it appears that it is not uncommon for people to travel unvaccinated to HAV-endemic countries. An outbreak investigation of hepatitis A in travellers to Egypt between 2012 and 2013 found a high proportion of travellers who were not immunised before travelling [16,17]. Interviews with the German cases have rendered similar results, suggesting that there may be an information gap regarding both the risk of hepatitis A and the availability of a safe and effective vaccine.

## Conclusions

Thus, vaccination recommendations for hepatitis A need to be repeatedly emphasised, particularly before and during peak travel seasons. In order to better reach individuals travelling to endemic countries, we propose engaging travel companies and airlines to inform and remind travellers about vaccination recommendations, as has been suggested by others [16,18]. Special attention should also be given to populations with a Moroccan origin living in the EU/EEA, who may regularly be travelling to Morocco. These populations need to be alerted that particularly their children are most likely susceptible to HAV infection and should be vaccinated [19]. Finally, as hepatitis A vaccination does not protect from many other food-borne infections additional recommendations to follow sound hygiene including hand hygiene should be given to the public.
